# Genetic circuits to engineer tissues with alternative functions

**DOI:** 10.1186/s13036-019-0170-7

**Published:** 2019-05-03

**Authors:** C. P. Healy, T. L. Deans

**Affiliations:** Department of Biomedical Engineering, University of Utah, Salt Lake City, UT 84112 USA

## Abstract

Persistent and complex problems arising with respect to human physiology and pathology have led to intense investigation into therapies and tools that permit more targeted outcomes and biomimetic responses to pathological conditions. A primary goal in mammalian synthetic biology is to build genetic circuits that exert fine control over cell behavior for next-generation biomedical applications. In pursuit of this, synthetic biologists have engineered cells endowed with genetic circuits with sensor that are capable of reacting to a variety of stimuli and responding with targeted behavior. Here, we highlight how synthetic biology approaches are being used to program cells with novel functions for therapeutic applications, and how they can be used in stem cells to improve differentiation outcomes. These approaches open the possibilities for engineering synthetic tissues for employing personalized medicine and to develop next-generation biomedical therapies.

## Introduction

Synthetic biologists use bottom-up approaches to assemble genetic parts into more complex gene circuits to enable the programming of new functions into cells. This approach combines individual gene expression parts, or modules, that can be characterized independently and used to build novel genetic circuits by combining multiple modules that interact with each other to perform a defined function in cells. The inception of synthetic biology started with engineering prokaryotes with novel functions [1–18], and efforts to engineer mammalian cells soon followed. Mammalian synthetic biology has traditionally focused on transcriptional and post-transcriptional regulation to program cells with new functions. These efforts include programming feedback [19–21], controlling gene expression levels [22–32], implementing Boolean logic functions [33–39], and targeting specific disease states [40–46]. More recent approaches that target specific locations in the genome using zinc finger (ZF) proteins, transcription activator-like effectors (TALEs), and clustered regulatory interspaced short palindromic repeats (CRISPR) coupled with a modified Cas9 protein with its nuclease activity removed (dCas9), have been used to interrogate endogenous transcription factors [47–56]. These studies have enabled the interrogation of endogenous DNA sequences to better understand the role of natural transcriptional networks within cells to better understand how cells control these networks [57, 58]. The details of these genetic tools have been extensively reviewed elsewhere [59–63], therefore, here we aim to provide a framework for using genetic circuits to design new therapies by engineering tissues with alternative functions.

Pluripotent stem cells are cells that have the potential to produce any cell or tissue in the body.

In the early development of complex organisms, pluripotent stem cells undergo specialized decision-making in a remarkably ordered process to yield tissue patterns, morphogenesis, and organogenesis [64–68]. The underlying mechanisms of this lineage specification process is not fully understood. However, coordinated clusters of transcription factors, or gene networks, have emerged as key regulators of stem cell pluripotency and differentiation. Additionally, studies have shown that dysregulation of these natural gene networks contributes to the onset of cancer and tissue degeneration, thereby underlying multiple types of human disease.

Stem cells can naturally direct their lineage commitment by controlling the timing and level of expression of key transcription factors resulting in desired differentiation pathways [69]. Previous work to recapitulate transcription factor expression in stem cells to drive differentiation into desired lineages included the overexpression of key transcription factors [70–73]. These studies demonstrated improved desired differentiation outcomes, however, this method often produces inefficient cell yields, relies on subpopulation selection, and generates heterogeneous cell types [74]. These challenges have recently led to efforts from synthetic biologists to implement genetic circuits capable of tight gene control that provide precise spatial and temporal expression of key transcription factors in stem cells.

In this review, we provide a framework for implementing synthetic biology in stem cells to direct stem cell differentiation into desired lineages. We detail studies that have implemented genetic circuits in stem cells and discuss the outcomes of these studies on the robustness of driving stem cell fate decisions. We next consider using synthetic biology to design artificial tissues that are endowed with alternative functions to provide new therapies for diseased states.

### Stem cells and synthetic biology

Stem cells play an important role in the development and regeneration of human tissues. A universal network of endogenous transcription factors control cell fate and continuously send and respond to physiological signals that adjust their cell-type specific gene expression. For example, the overexpression of the master transcription factors Oct4, Sox2, Klf4, and c-Myc is capable of overriding previously made cell fate choices to convert somatic cell types into a pluripotent state [75–81]. However, the differentiation of pluripotent precursor cells into adult cell types requires tightly controlled spatial and temporal gene expression dynamics of lineage-specific master transcription factors. Stem cell differentiation and the development of organs involves a complex coordination of both intrinsic and extrinsic cues that control cell behavior. This coordination of cues is critical for stem cells to make fate decisions and for robust tissue to develop.

**Fig. 1:**
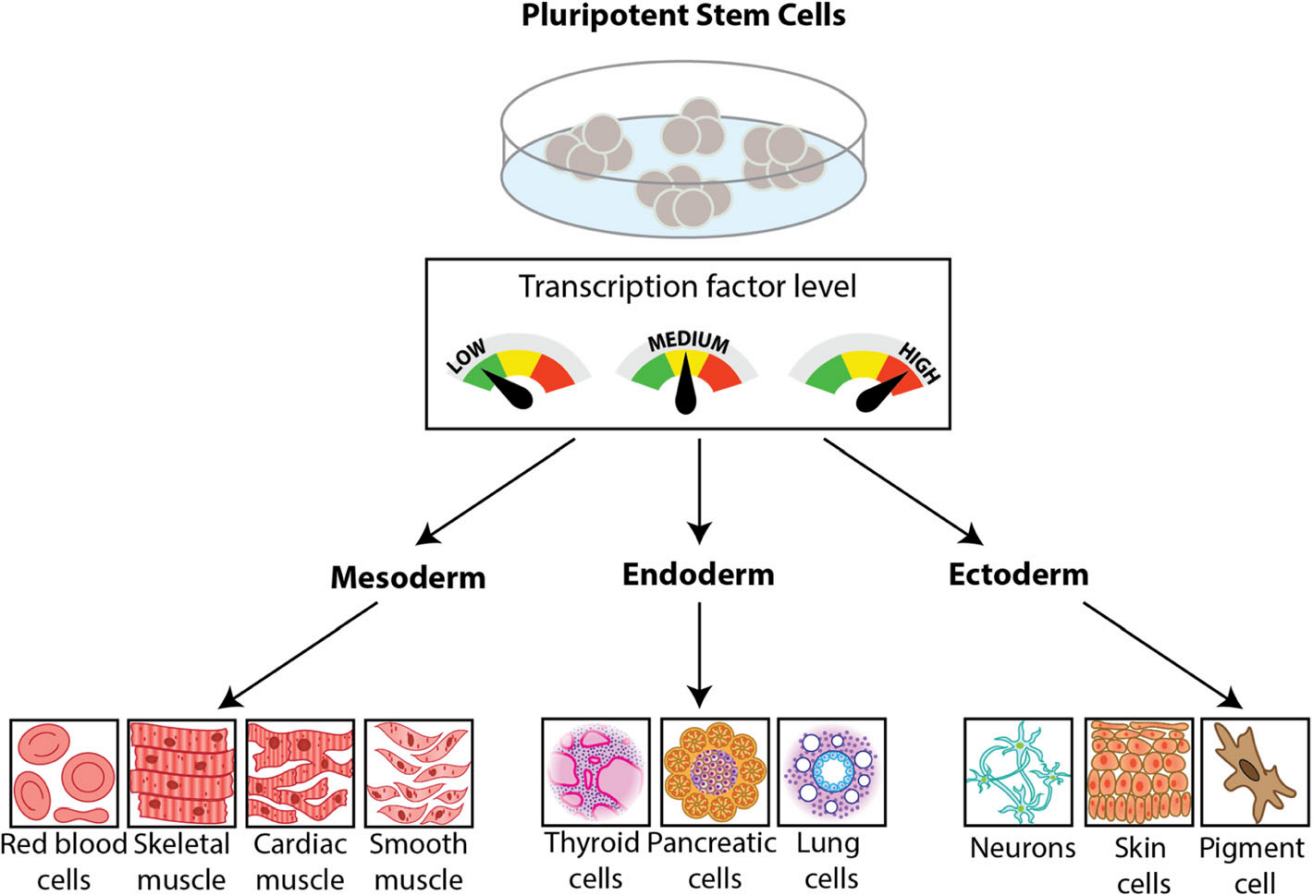
Tools in synthetic biology to drive stem cell differentiation. Genetic tools built by synthetic biologists enable tight control of gene expression that allow the dynamic control of transcription factor expression in stem cells. This includes various levels of expression (e.g. low, medium, and high), in addition to controlling levels of expression in dynamic patterns including tuning, pulsing, oscillations, etc. Controlling the levels, timing, and patterns of gene expression at various checkpoints of stem cell fate improves differentiation outcomes

Significant efforts are currently underway to program stem cells with genetic circuits to push their differentiation into desired lineages. Implementing genetic circuits to dynamically control gene expression (e.g. transcription factor expression) in stem cells is thought to improve differentiation outcomes because these circuits are able to replicate the dynamic gene expression patterns that are observed during development. Recently, a new genetic circuit was constructed coupling genetic parts from a mold, *Neorospora crassa,* and the bacterial Lac repressor system to create an orthogonal genetic switch to be used in mammalian cells [27]. After confirming tunability in immortalized cell lines for proof of concept performance, the tight gene control and tunability of gene expression of this genetic switch were demonstrated in pluripotent stem cells. These results suggest that synthetic biologists can program stem cells with artificial decision-making abilities that can be used to direct stem cell fate into desired lineages. For example, using genetic circuits that control the level and timing of expression of multiple transcription factors, it is possible to tune key cell fate regulators at various differentiation checkpoints to drive the differentiation of stem cells into one or many desired cell fates (Fig. 1).

To demonstrate the utility of using genetic circuits to drive decision-making in cells, a two-way communication genetic circuit was engineered to mimic the natural gene expression patterns during angiogenesis, the formation of blood vessels [82]. Cell-to-cell communication was the framework for this synthetic network. Specifically, sender cells were programmed with a genetic circuit to constitutively express tryptophan synthase (TrpB^26^), an enzyme from *E. coli*, which converts indole (a compound in the media) into L-tryptophan. The authors also engineered receiver cells with a genetic circuit designed to allow the cells to sense the secreted L-tryptophan and, in response, turn on the expression of a reporter gene, secreted alkaline phosphatase (SEAP). Next, the authors used this engineered cell-cell communication system to implement enhanced angiogenesis. During natural angiogenesis, two transcription factors, vascular endothelial growth factor (VEGF) and angiopoietin-1 (Ang1), function in a sequential and coordinated fashion to produce mature blood vessels [83]. In the engineered cell-cell communication system, the sender and receiver cells each possessed genetic circuits that generated output genes expressed at different times, to guide cellular differentiation and produce blood vessels. Due to the relatively small diffusion length of nutrients into tissues, vascularization strategies, such as angiogenesis in newly formed tissue, will be critical to the success of engineered tissues.

Genetic circuits have also been used to program stem cells with decision-making capabilities that enable them to produce efficient numbers of beta (β) cells. β cells are the cells found in the pancreas that synthesize and secrete insulin in response to glucose in the blood in a dose-dependent manner. Type 1 diabetes is a chronic condition in which the pancreas produces little to no insulin, and the primary cause of Type 1 diabetes is believed to be an auto-immune destruction of the β cells. The resulting destruction of these cells reduces the body’s ability to respond to glucose levels, making it nearly impossible to regulate glucose levels in the bloodstream properly. To develop alternative therapies for Type 1 diabetes, scientists have focused on producing β cells in vitro from pancreatic progenitor stem cells by overexpressing the three master-regulator transcription factors, Pdx1, Ngn3, and Mafa. This approach results in the differentiation of pancreatic progenitor stem cells into mature insulin producing β cells [76, 84]. Recently, a genetic circuit that functions as a band-pass filter was built to dynamically control the expression of the three master-regulator transcription factors [85]. This genetic circuit enabled the timely coordination of the three transcription factors, which produced a homogeneous population of cells that demonstrated robust insulin production over cells produced using traditional growth factor and chemical based techniques. This study emphasizes the need for the temporal regulation of gene expression during cell fate decisions.

In addition to controlling when key transcription factors turn on during differentiation, a recent study has shown that some cell fate pathways require pulsing the expression of key transcription factors [72]. In this study, the pulsing expression of Gata6 in human induced pluripotent stem (iPS) cells initiated the formation of all three germ layers giving rise to a complex three-dimensional (3D) multicellular tissue construct, or organoid, that exhibited a liver bud-like phenotype. Without pulsing only one germ layer formed. Genetic circuits enable fine-tuned control over the expression of transcription factors, suggesting that the possible gene expression patterns that can be implemented using genetic circuits are effectively limitless. Patterns including pulsing, tuning, oscillations are all within the realm of possibilities. Therefore, genetic circuits offer extraordinarily precise control over gene expression and cell fate that will likely transform their applicability in basic science and clinical research.

### Replicating physiological functions in alternative cell types

Precise control over the intensity, duration, and timing of gene expression have advanced our abilities to direct stem cell fate into desired lineages, in addition to developing organoids. Using the same genetic tools, synthetic biologists have also created therapeutic cells that are capable of sensing and responding to various signals in a therapeutic fashion [43–45, 86–104]. For example, in two separate studies, genetic circuits were used to regulate glucose levels in the bloodstream of diabetic mice. In the first study, the expression and secretion of the glucagon-like peptide 1 (GLP-1), a peptide that has the ability to decrease blood sugar levels in the blood by enhancing to secretion of insulin [105], was controlled in human embryonic kidney (HEK) 293 cells using an optogenetic-controlled genetic circuit [106]. This genetic circuit allowed the implanted cells to detect blue light and, in response, initiate the transcription of GLP-1, causing blood glucose levels to fall in diabetic mice. In the second study, Chinese hamster ovarian (CHO) cells were engineered with a genetic circuit that produced insulin in response to decreasing pH levels. This study demonstrated controlled production of insulin when environmental pH dropped below the physiological range [41]. When these engineered cells were implanted into diabetic mice, they were able to restore insulin and glucose levels to the same level as that of healthy mice.

In addition to engineering therapeutic cells for metabolic disorders, a recent study demonstrated the use of a genetic circuit to endow HEK293 cells with the ability to control an inflammatory response [107]. This circuit was comprised of three basic modules to detect and respond to inflammatory signals: a sensor to sense inflammation signals; an amplifier with positive feedback to ensure sustainability of the response; and an effector that neutralized the inflammatory response. Therapeutic cells that are endowed with the ability to keep the body’s inflammatory response in check are an exciting advance in the field because these types of cells can be implanted after surgery to prevent a prolonged inflammatory response from hindering may proper healing and to allow for the restoration of healthy levels of inflammatory cytokines.

### Future directions

Mammalian synthetic biologists have made great strides in engineering novel genetic tools to tightly regulate gene expression in various cell types. These genetic tools have been used for directing stem cell differentiation to produce desired cell lineages, to make organoids, and to engineer therapeutic cells to sense and respond to disease. With these accomplishments under our belts, it stands to reason that synthetic biologists can engineer implantable mini tissues, or organoids, that have been engineered to sense and respond to disease.

**Fig. 2:**
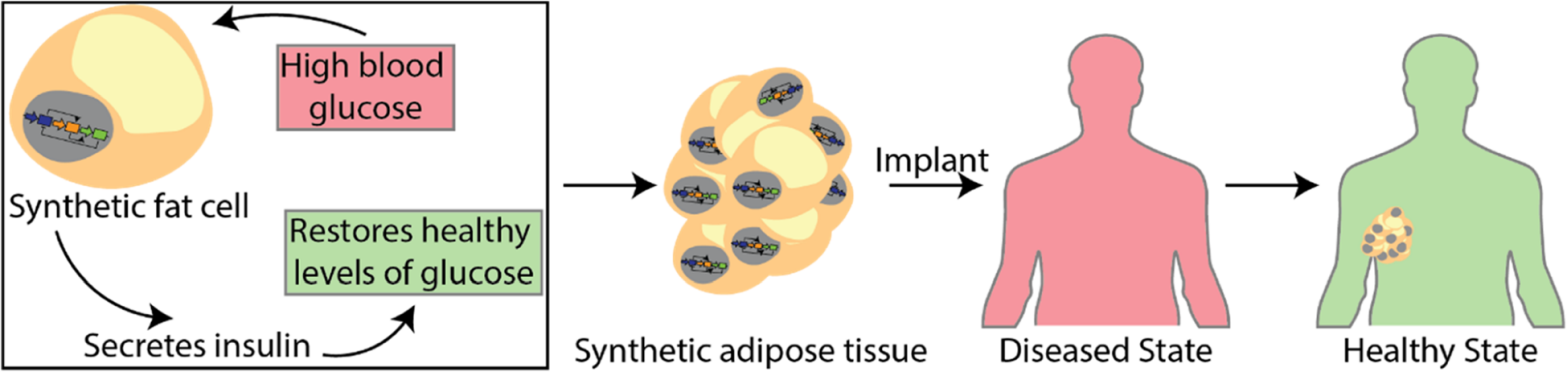
Engineering synthetic tissues for employing personalized medicine. Engineering synthetic fat tissue for regulating blood glucose levels in diabetic patients will likely be a reality in the near future. In this hypothetical therapy, adipose cells are programmed with a genetic circuits capable of sensing glucose levels and, if pre-programmed physiologically high levels of glucose are sensed, they will respond by secreting a tightly regulated amount of insulin. These small synthetic tissues can be implanted under the skin in unnoticeable locations (e.g. in the armpit), to regulate blood glucose levels over long periods of time

Rather than trying to recreate a failing pancreas for diabetic patients, can we engineer implantable adipose tissue (fat) with the ability to regulate blood glucose levels? Studies have shown that a patients’ own fat can be harvested and injected back into the individual’s joints to help alleviate joint pain [108]. In fact, Lipogems are an FDA approved system that are small bits of fat removed individuals, washed, and reinjected into various joints for those suffering from spinal conditions, joint pain, arthritis, or rotor cut tears [109–112]. One can start to image engineering personalized synthetic adipose tissue by first making iPS cells from a patient’s skin cells, and programming them with various genetic circuits: one to drive the differentiation into adipose cells, and the other with a program to regulate blood glucose levels (Fig. 2). These small synthetic tissues can then be injected under the armpit, or other unnoticeable locations, to regulate blood glucose levels over long periods of time. Of course, once one accepts the idea of injectable synthetic fat tissue, it’s easy to imagine engineering other synthetic organs (e.g. skin) that can function in various ways to improve the health of an individual.

Organoids are another area where synthetic biologists can have a significant impact. Unlike a purified tissue, like adipose tissue, organoids are miniaturized versions of an organ that can be isolated from organ progenitor cells, or pluripotent stem cells, and differentiated to form an organ-like structure. These organoids have multiple cell types that self-organize to create a structure similar to an organ found in vivo [113]. Because multiple cell types are in organoids, this offers opportunities to engineer more complex interactions (e.g. synthetic pattern formation, spatial and temporal communication between cell types, etc.) that may be required to recapitulate failing organs whose function cannot be replaced with a single tissue. For example, a synthetic organoid can be engineered with genetic circuits that can mimic healthy pathways to alter underlying disease states and rewire them to restore the healthy state [114].

## Conclusion

Engineered therapeutic cells that are endowed with genetic circuits have the potential to transform basic science and medicine. Using genetic circuits to tightly control the expression of transcription factors has shown to significantly improve differentiation outcomes. With the improvements to controlling gene expression in cells that continue to be built by synthetic biologists, we will continue to push the envelope of cell engineering possibilities. Altogether, these efforts will result in the rapid and precise engineering of cells, tissues, and organoids that will lead to transformative clinical applications.
